# Introduction of a section for recording dementia improves data capture on the ambulance electronic patient record: evidence from a regional quality improvement project

**DOI:** 10.29045/14784726.2024.9.9.2.29

**Published:** 2024-09-01

**Authors:** Phil King, Patryk Jadzinski, Helen Pocock, Chloe Lofthouse-Jones, Martina Brown, Carole Fogg

**Affiliations:** South Central Ambulance Service NHS Foundation Trust ORCID iD: https://orcid.org/0000-0001-7736-7183; South Central Ambulance Service NHS Foundation Trust; University of Portsmouth ORCID iD: https://orcid.org/0000-0002-6752-0807; South Central Ambulance Service NHS Foundation Trust ORCID iD: https://orcid.org/0000-0001-7648-5313; South Central Ambulance Service NHS Foundation Trust ORCID iD: https://orcid.org/0000-0001-8118-3934; South Central Ambulance Service NHS Foundation Trust ORCID iD: https://orcid.org/0000-0003-3083-8958; University of Southampton ORCID iD: https://orcid.org/0000-0002-3000-6185

**Keywords:** dementia, electronic medical record, older adults

## Abstract

**Introduction:**

Dementia is a common co-morbidity in older people who require urgent or emergency ambulance attendance and influences clinical decisions and care pathways. Following an initial audit of dementia data and consultation with staff, a specific section (tab) to record dementia was introduced on an ambulance service electronic patient record (ePR). This includes a dementia diagnosis button and a free-text section. We aimed to assess whether and how this improved recording.

**Methods:**

To re-audit the proportion of ambulance ePRs where dementia is recorded for patients aged ≥65 years, and to describe the frequency of recording in patients aged <65; to analyse discrepancies in the place of recording dementia on the ePR by comparing data from the new dementia tab and other sections of the ePR.

**Results:**

We included 112,193 ePRs of patients aged ≥65 with ambulance attendance from a six-month period. The proportion with dementia recorded in patients aged ≥65 was 16.5%, increasing to 19.9% in patients aged ≥75, as compared to 13.5% (≥65) and 16.5% (≥75) in our previous audit. In this audit, of the 16.5% (n = 18,515) of records with dementia recorded, 69.9% (n = 12,939) used the dementia button and 25.4% (n = 4704) recorded text in the dementia tab. Dementia was recorded in ePR free-text fields (but not the dementia tab) in 29.7% of records. Eighteen other free-text fields were used in addition to, or instead of, the dementia tab, including the patient’s social history, previous medical history and mental health. Dementia was present on the ePR of 0.4% (n = 461) of patients aged <65.

**Conclusions:**

An ePR dementia tab enabled ambulance clinicians to standardise the location of recording dementia and may have facilitated increased recording. We would recommend other ambulance trusts capture this information in a specific section to improve information sharing and to inform care planning for this patient group.

## Introduction

Older adults are frequent users of ambulance services in the UK. In the South Central region, 48% of 999 calls in 2022/2023 were for adults aged ≥65, equating to approximately 21,200 calls per month. Dementia is a common co-morbidity in older adults, with an estimated 900,000 people in the UK currently living with dementia, projected to increase to 1.6 million by 2040 (Alzheimer’s Society, 2023). Importantly, around 61% of people with dementia live at home rather than in a residential care setting and are supported by either unpaid or paid-for care at home (Social Care Institute for Excellence, 2023).

Whatever the reason for calling an ambulance, knowledge of concurrent dementia or other cognitive impairments can assist clinical staff to provide person-centred care on scene and to determine the most appropriate onward care pathway (Bernard et al., 2021). It is well recognised that hospital admissions can lead to significant deterioration for people with cognitive impairments (Fogg et al., 2017, 2018), and that other forms of care either in, or closer to, the person’s home, such as urgent community response visits, advanced illness management programmes led by community paramedics or anticipatory care planning, may be of more benefit (Abrashkin et al., 2016; Björck & Wijk, 2018; British Geriatrics Society, 2021; Buswell et al., 2016; Evans, 2011). Consistent recording of dementia could therefore facilitate easier information retrieval for other care providers, for example those with access to an electronic summary care record, and could also assist future emergency attendances to speed up assessment and decision making.

**Figure 1:**
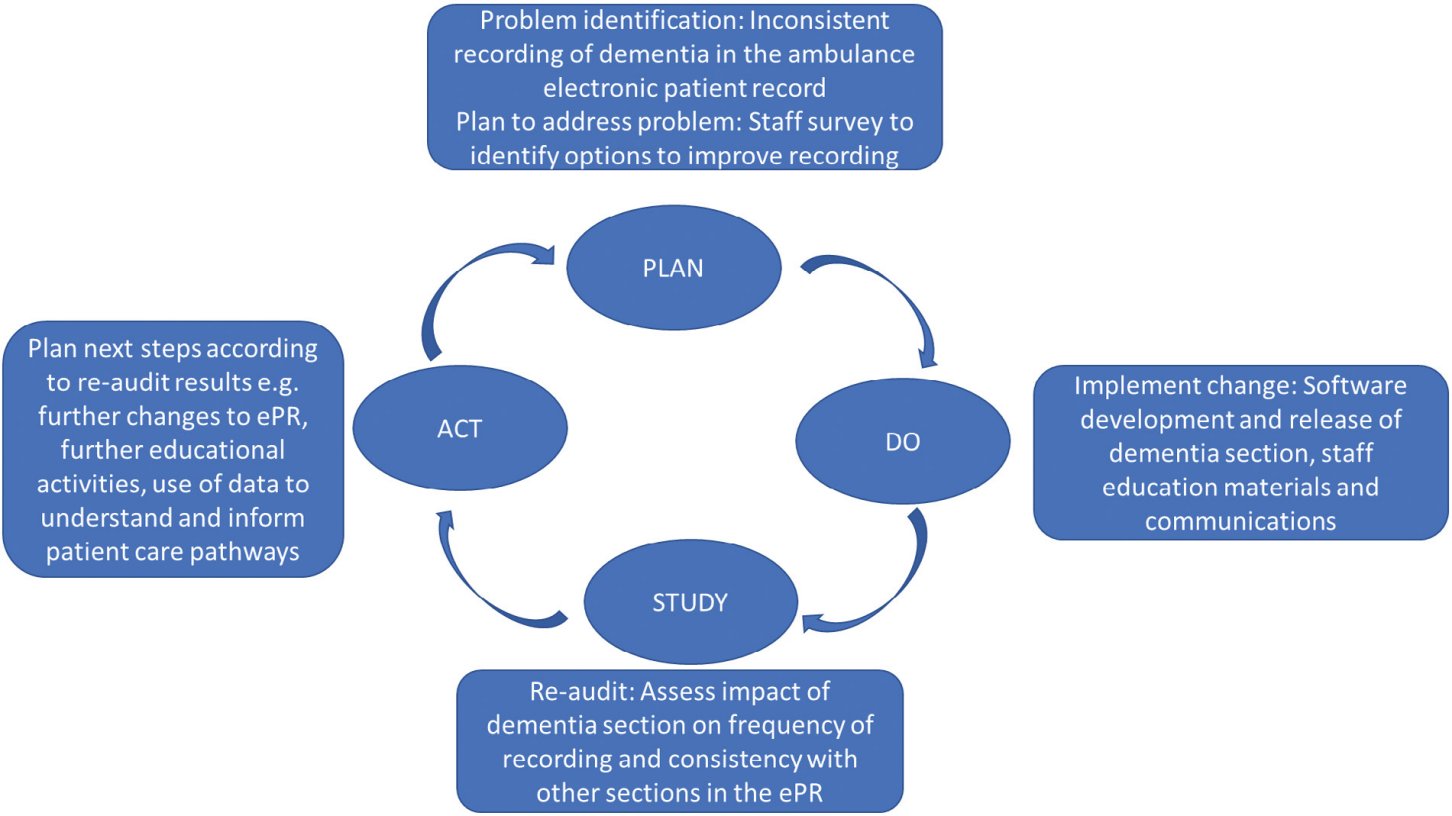
Figure 1. Plan-Do-Study-Act cycle for a quality improvement project focusing on improving capture of dementia within the ambulance electronic patient record.

Our previous audit of the electronic patient records (ePRs) of a regional ambulance service with no specific area in which to record dementia found that dementia was recorded in 16 different free-text fields across various sections in the ePR and that 38.4% of these records had dementia recorded in more than one place (Pocock et al., 2018). We initiated a quality improvement project, which began with a staff survey (the IDEAS survey) to understand how dementia was identified and recorded on the ePR, and found many staff would prefer to have a designated section or menu ‘tab’ (Jadzinski et al., 2021). Following further consultation with clinical staff and members of the public with an interest in the care of people with dementia, we developed and implemented a section for the ePR to facilitate better recording as a step within a plan-do-study-act (PDSA) quality-improvement cycle (Taylor et al., 2014) (Figure 1). Here we report the findings of a re-audit of the data following introduction of the dementia tab as part of the ‘study’ phase of the cycle.

### Objectives

Our study had three main objectives:

1. To perform a re-audit to assess the proportion of ambulance ePRs where dementia is recorded for patients aged ≥65 years and to describe the frequency of recording in patients aged <65;
2. To quantify and describe discrepancies between the new dementia section and other free-text ePR sections and dementia recorded as a presenting complaint;
3. To describe conveyance and referral patterns stratified by recording of dementia.

### Audit standard

The audit standard was consistent recording of a diagnosis of dementia (where present and relevant) on the patient’s health record (Skills for Health, 2018).

### Sample

Anonymised ePRs of patients aged ≥65 and patients aged <65 dating from October 2022 to March 2023 inclusive with any field indicating dementia were identified. For the main analysis, records from the following patient attendances were included:

1. Patient aged ≥65 years;
2. An electronic record with a date and time of attendance available between 1 October 2022 and 31 March 2023;
3. Attendance by emergency ambulance clinicians, including registered paramedics, nurses or doctors, ambulance technicians and associate ambulance practitioners;
4. Those within a clinical commissioning group area with ≥70% electronic records available (i.e. reflecting areas staffed mainly by South Central Ambulance Service NHS Foundation Trust (SCAS) crews rather than private providers).

Records for patients who were known to be deceased at the time of ambulance arrival on scene and visits from patient transport services were excluded. Additionally, aggregate data of dementia frequency (defined as dementia being recorded anywhere in the ePR) in five-year age bands were extracted across all ages.

### Data source

The study focused on patients with emergency ambulance attendances within the geographical boundaries of the SCAS, including Hampshire, Berkshire, Buckinghamshire and Oxfordshire, which serves more than four million people.

## Methods

### Data input and extraction

Electronic patient records are created at the scene by emergency ambulance staff using the MobiMed Smart electronic tablet (Ortivus, Sweden). The ePR system is used to collect patients’ clinical and social history, incident details, clinical information and decisions on conveyance and referral. Data are entered into the ePR via a touchscreen on a tablet, through menus and interactive, self-expanding boxes, as well as sections of free text for additional detail about the examination where necessary. Data are transferred to a warehouse and downloaded daily to the SCAS Business Intelligence Team. Microsoft SQL Server Management Studio was used to extract data from the SCAS data warehouse. Records with the term ‘dementia’ entered in the ePR were identified using queries of free-text fields, as described previously (Pocock et al., 2018). The ePR dataset was anonymised, uploaded into a standalone application and matched with incident numbers using computer-aided dispatch (CAD) information to link to the triage grade. A random sample of free text, where the word ‘dementia’ has been written in a field that is not in the dementia section, was extracted.

### Description of software changes

**Figure 2:**
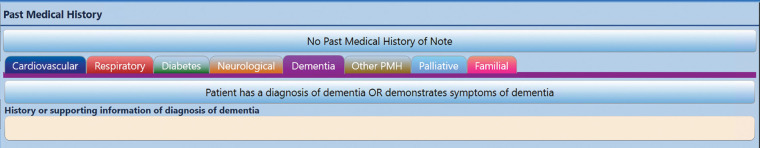
Figure 2. Ortivus electronic patient record data entry system displaying new dementia tab.

Following the outcome of the first audit and the IDEAS survey (Jadzinski et al., 2021), the Ortivus ePR system was updated to include two additional dedicated fields for the recording of dementia on a separate tab in the past medical history section, labelled ‘Dementia’ (Figure 2). The first field was a ‘button’ labelled ‘Patient has a diagnosis of dementia OR demonstrates symptoms of dementia’. Selection of this button by the ePR user indicates that clinicians have evidence that the patient has a diagnosis of dementia or they suspect that the patient may have dementia. The second field is free text, which allows clinicians to record their reasoning or supportive information. The fields are independent of each other, that is, clinicians can complete one, both or none.

The software changes were part of a system-wide update to the Ortivus system that took place in October 2022. Communications were sent to all ePR users once in the week prior to the release of the update. Clinical staff were informed of the change using targeted messages and notifications via SCAS internal communication channels including the ‘Staff Matters’ electronic newsletter, social media channels ‘Research’ Yammer and ‘SCAS’ Yammer, information via operational teams management (Clinical Team Educators / Team Leaders) and via the Education Team. Within three days of the update being available, 98% of all Ortivus devices had received the new fields.

In the software release that included the dementia tab, ‘Dementia’ was also added to the drop-down list in the presenting complaint data field (labelled ‘Impression or Suspected Diagnosis’ in the ePR), which is a place for clinicians on scene to record the main reason for treating the patient or the highest-priority issue for the call.

### Data analysis

Descriptive statistics were calculated for the total number of emergency attendances and the proportion of conveyances and were summarised according to a record of dementia anywhere in the ePR. The number of records with agreement and discrepancy between the dementia section and other areas of the ePR in which dementia was recorded was calculated. Patient and clinical characteristics and types of referrals were summarised for incidents with and without a record of dementia for patients aged ≥65 years. Free-text fields discrepant with the dementia tab were summarised qualitatively.

### Patient and public involvement

The need for this work was identified by the SCAS Patient Forum, who were concerned that sometimes their family, friends or neighbours were taken to hospital due to lack of alternative care, and that once in hospital, they took a long time to return home. Two online sessions were held with public contributors who identified specific needs for people with dementia requiring urgent care and the importance of identifying and managing those needs at the time of ambulance attendance. The results of the evaluation will be shared with these groups, who will assist with dissemination to a public audience and will help define next steps.

### Approvals

The audit was approved by the SCAS Clinical Review Group on 20 October 2022. All data were anonymised. The large dataset and group sizes used for analysis further preclude unintended identification of any individuals.

### Caveat

It is not mandatory to complete the dementia tab for all patients. Absence of recording of dementia in the tab or elsewhere on the ePR does not rule out that the patient may have dementia, but infers that these data were not considered relevant to the incident.

## Results

### Description of included records

There were 225,066 Ortivus records during the six-month audit period, with 49.8% (n = 112,193) associated with patients aged ≥65 years.

### Uptake of dementia tab

Saturation of field usage (i.e. an increase in use followed by a plateau) was achieved within the first few days of the dementia tab being available to clinicians to use on the ePR (Supplementary 1).

### Recording of dementia

There were 19,007 ePRs with dementia recorded, of which 97.4% (n = 18,515) were for patients aged ≥65 years. For these patients, the dementia button in the dementia tab was used in 69.9% (n = 12,939) of records, and 36.4% (n = 8235) of records with the button selected also had further details in the dementia tab free text (Table 1). The free-text field in the dementia tab was only completed when the dementia button was selected.

**Table 1.** Summary of recording in the dementia tab for patients aged ≥65.

Dementia button selected?	Dementia tab free-text field includes ‘dementia’?		Total
	*Yes*	*No*	
*Yes*	4704	8235	12,939
*No*	0	99,254	99,254
**Total**	4704	107,489	112,193

Other areas where dementia was recorded include the ‘Impression/suspected diagnosis’ field in 2.9% (n = 540), and free-text areas on the ePR (but not in the dementia tab) in 29.7% (n = 5503). However, inconsistencies between the dementia tab and the presenting complaint field and other free-text fields in the ePR were observed (Table 2).

**Table 2.** Recording in the dementia tab as compared to other ePR fields.

Patients aged <65				Patients aged ≥65			
	Dementia button ticked?			Dementia button ticked?			Grand total
	Yes	No	Total	Yes	No	Total	
**Impression/diagnosis detail is ‘Dementia’**							
Yes	5	12	17	337	203	540	557
No	251	112,605	112,856	12,602	99,051	111,653	224,509
**Total**	**256**	**112,617**	**112,873**	**12,939**	**99,254**	**112,193**	**225,066**
**Dementia in free-text fields^a^**							
Yes	146	177	323	7952	4173	12,125	12,448
No	110	112,440	112,550	4987	95,081	100,068	212,618
**Total**	**256**	**112,617**	**112,873**	**12,939**	**99,254**	**112,193**	**225,066**

^a^Does not include free-text field in the dementia tab.

For patients aged ≥65 with the ‘Impression/diagnosis’ field indicated, only 60.5% (n = 337) also had the dementia tab completed. There were 18 free-text fields where dementia was recorded (other than the field on the dementia tab), including the patient’s social history, presenting complaint, medical and previous medical history text, examination details and mental health. In the 12,448 records where this occurred, 63.9% (n = 7952) also had the dementia tab completed.

For a sample of records where the dementia tab was not completed but ‘Dementia’ was recorded in other ePR free-text fields, the majority of free-text entries referred directly to the patient as having dementia, and a minority referred to the patient having a suspected undiagnosed dementia in the view of their family and/or the ambulance crew. However, discrepant records with free-text entry in the ‘social history’ free-text field included descriptions of the attended patient being the main carer for a husband or wife with dementia and, in some cases, a record that both the patient and their spouse had dementia.

Analysis of the records with dementia recorded in people aged between 40 and 64 years showed 17 records (0.02%) with dementia as the ‘Impression/diagnosis’, 256 (0.23%) with the dementia tab button selected and 146 (0.13%) with further information in the dementia tab free-text field.

### Characteristics of patients aged ≥65 according to a record of dementia

**Figure 3:**
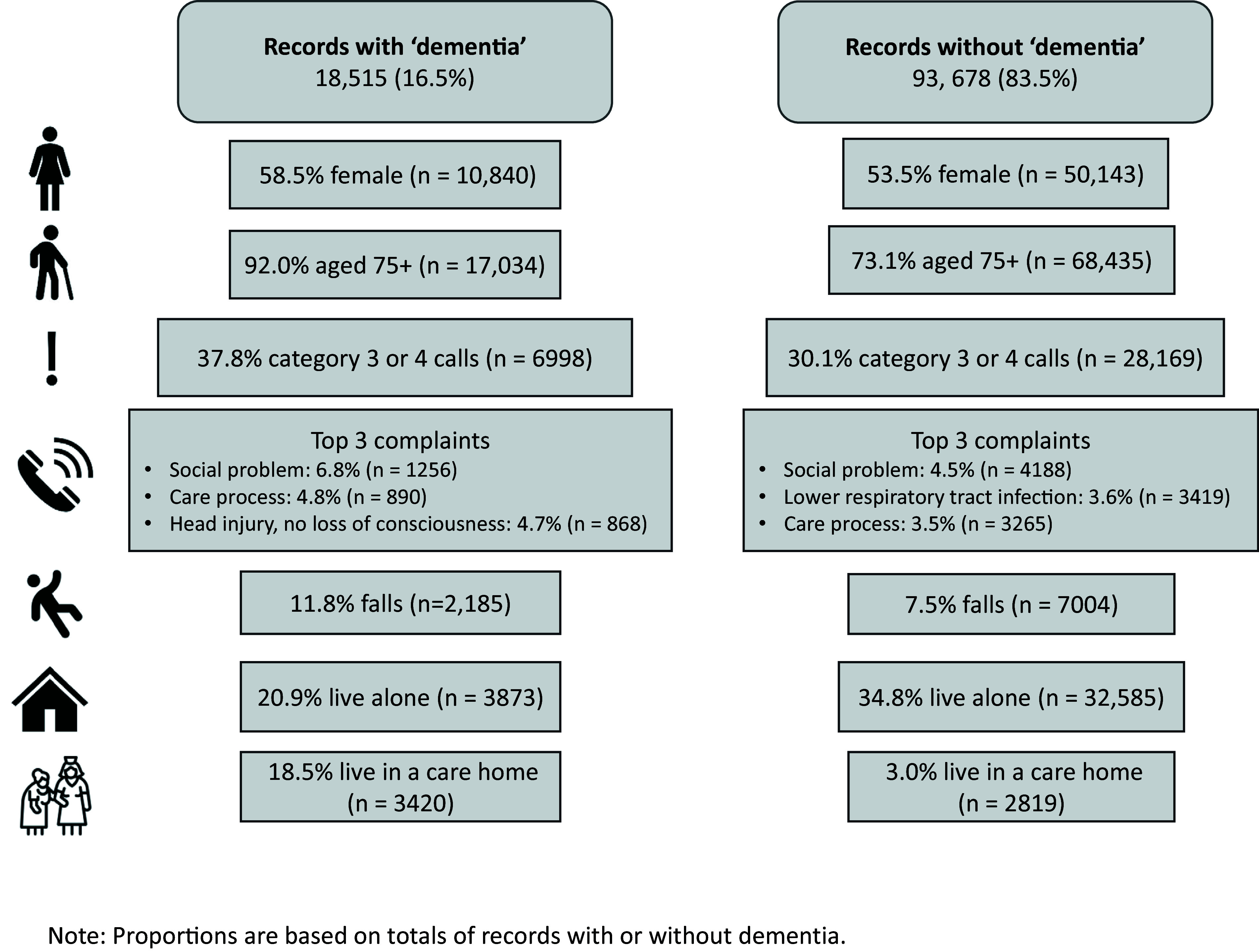
Figure 3. Overview of patient and incident characteristics.

Patients aged ≥75 comprised 76.2% (n = 85,469) of all attendances to patients aged ≥65. Dementia was recorded in either the dementia tab or elsewhere in the ePR for 16.5% (n = 18,515) of attendances, increasing to 19.9% (n = 17,034) in patients aged ≥75 years. Patient and incident characteristics are displayed in Figure 3. Incidents with a record of dementia had a higher proportion of patients aged ≥75, more females and a higher proportion of category 3 or 4 calls (urgent but not life-threatening). The most common presenting complaints were similar, although trauma was the third most common in patients with dementia, as compared to respiratory infections being second in those without, and falls were also more common in those with dementia. The proportion of patients living alone was lower in those with dementia, perhaps reflecting a larger number of attendances to residential care in this group.

### Attendance outcomes according to dementia recording

The conveyance rate for patients aged ≥65 overall was 66.1% (n = 74,119), with 68.5% (n = 18,307) of patients aged 65–74 conveyed, and 65.3% (n = 55,812) of patients aged ≥75 years. Conveyance rates in patients with a record of dementia anywhere in the ePR were 61.9% (n = 917) and 59.8% (n = 10,190) for patients aged 65–74 and ≥75, respectively, as compared to 68.9% and 66.7% in those without a recording of dementia. Non-hospital alternative care pathways for people aged ≥65 were used in 3.6% of attendances overall, but for 5.1% of attendances with a dementia record (Table 3).

**Table 3.** Non-hospital care pathways for patients with and without dementia in the ePR.

Referral pathway	Dementia record in ePRs for patients aged ≥65		
	Yes n = 18,515	No n = 93,678	Total n = 112,193
General practice	317 (1.7%)	1225 (1.3%)	1542 (1.4%)
Other community services	277 (1.5%)	834 (0.9%)	1111 (1.0%)
Out-of-hours services	158 (0.9%)	456 (0.5%)	614 (0.5%)
Falls service	123 (0.7%)	381 (0.4%)	504 (0.4%)
District nurse	63 (0.3%)	184 (0.2%)	247 (0.2%)
**Total**	**938 (5.1%)**	**3080 (3.3%)**	**4018 (3.6%)**

## Discussion

### Observations

The inclusion of a dementia tab on the ambulance ePR has improved standardisation in the recording of dementia, with the majority of incidents where dementia is recorded resulting in the tab being used rather than only using other free-text fields. Compared to the previous audit, the proportion of incidents with dementia recorded for people aged ≥75 increased from 16.5% (Pocock et al., 2018) to 19.9%. This increase is likely to reflect the changes in the ePR, which facilitate easier recording and should consequently improve ease of retrieval of information by clinicians who often re-attend this patient group.

The majority of incidents with dementia recorded referred to adults aged ≥65; however, more than 200 incidents in middle-aged adults had dementia noted by the clinician on scene, highlighting awareness of the condition in younger adults. In some of these cases, dementia related to a spouse or family member rather than the patient themselves, indicating that consideration of the wider familial and caring situation is key to understanding decisions made in the emergency setting. Therefore, use of other ePR sections to record dementia is still necessary to be able to describe these situations appropriately.

The conveyance rate is an important indicator of emergency care quality for people living with dementia, and this was consistent with the previous audit, rising slightly from 64.1% to 65.3% in people aged ≥75 and remaining lower than the conveyance rate for people without a record of dementia. Additionally, rates of referrals to non-hospital services (community services, out-of-hours and falls services) were slightly higher in people with dementia than in those without. Although expansion of services, including community urgent care response and frailty teams and other non-A&E same-day emergency care (SDEC) services (Elias et al., 2024), has occurred since the original audit, the stable conveyance rate suggests that there is more scope for improved access to currently available services to be able to potentially reduce conveyances. Further analysis of on-scene contexts from clinicians and patients/carers regarding their current and unmet needs will be pivotal in informing the tailoring of services to better meet urgent care needs and in avoiding unnecessary escalation of care in future (Abreu et al., 2019).

### Recommendations

Understanding the urgent care needs of older adults is key to informing development of care pathways and to identifying inequalities in access to care and subsequent outcomes. Increasing pressures within the health and social care systems related to reduced funding and workforce shortages necessitate better intelligence to inform service organisation to maintain good outcomes for patients. Decision making around common reasons for attendance to people living with dementia, such as falls or end-of-life care, is complex and likely to be affected by variability in local services (Halter et al., 2011; Waldrop et al., 2018). Having a specific dementia tab in the ePR enables improved monitoring of conveyance and referral rates, evaluation of communications with clinical staff and identification of required changes to care pathways. Likely benefits to patients include increased awareness of diagnosis or symptoms among staff who provide care further along the pathway, which should in turn improve person-centred care and patient outcomes.

Our data reinforce that there are large numbers of people with dementia requiring care from ambulance services and that a proportion of these incidents are mainly related to dementia rather than another co-morbidity, as indicated by the impression/suspected diagnosis field. Greater understanding of the nature of incidents where signs and symptoms of dementia are the main reason for the attendance, including those in which care may have been missing prior to the urgent care episode, and where the patient’s presentation influenced their onward care pathway should inform improvements in care for this large patient group.

We propose that all ambulance trusts have a specific place to record dementia as a common and significant co-morbidity in older people, in addition to considering including dementia in the presenting complaint field where it is the main reason for the call. Although this is probably unsuitable to be a mandatory field in the ePR, the use of a pop-up reminder window or similar alert for patients aged ≥65 could be considered. This should help with establishing needs for this patient group and, consequently, aligning localised design of services within integrated care systems related to the trust regions.

### Action plan

The question format on the button may be adapted into two separate questions to differentiate between people with an existing diagnosis and those who are symptomatic as observed by the clinician on scene or from carer/family reports. NHS digital data show that only 62% of patients aged ≥65 who are estimated to have dementia actually have a diagnosis in their primary care record (NHS Digital, 2023). However, it is likely that evidence of common symptoms of dementia will also influence outcomes of assessment and conveyance/referral decisions and is, therefore, important to record. Further examination of the social history free-text field to quantify how often the recording of dementia related to people other than the patient may necessitate either exclusion or better-adapted phraseology to include this field in future analysis.

This audit sits within a wider quality improvement project, which has also collected survey data from front-line staff regarding the use of the dementia tab, dementia education for staff, data collection and available referral services. Survey data will be used in conjunction with this re-audit information to interpret current care needs and patterns within the region and to identify areas for further exploration, including variations in demand, alternative care pathway availability and impact on patient pathways and outcomes. Evaluation of the impact of having this information on the ability of onward services, such as the emergency department, to provide prompt appropriate care and greater understanding of the decision-making process of the clinical team on scene will be explored.

### Limitations

The function of the ePR as a clinical recording device, rather than a research tool, means the ‘button’ used to indicate dementia only records ‘positively’ – that is, that there is evidence present – but it is not possible to distinguish between ‘not present’, ‘not known’ or ‘not recorded’. Therefore, incidents without dementia recorded cannot be assumed not to have dementia, and so this data must be interpreted accordingly. Clinicians will not necessarily assess each patient for dementia and our data may include multiple visits to an individual, so this is not the same as the prevalence of dementia in the population being attended, although it may provide a useful indicator of numbers and trends.

Factors other than the presence of the dementia section may have influenced the change in the proportion of ePRs with dementia recorded since the initial audit, including changes in rates of dementia diagnosis in the region, changes in dementia prevalence following high mortality rates in older people during the acute phase of the COVID-19 pandemic and additional staff training and awareness. However, the improvement in the consistency of recording suggests that a specific section has made it easier for staff to decide where to record dementia, which may have increased completeness of recording.

## Conclusions

Having a specific section to record dementia on the ambulance ePR aids standardisation of recording of information. This is key to capture more complete and accurate information given the large numbers of older people requiring emergency care and the complexity of assessment and decision making for the clinical team. Further feedback from staff and monitoring of future recording will enable continued refinement of the ePR.

## Acknowledgements

We would like to express our thanks to the members of the public who were involved in the development of the project. We would like to thank the SCAS staff that have engaged with the project and given input to the design of the ePR changes. We are grateful to the SCAS Education Technology team for preparing infographics to accompany the launch of the dementia tab and to Mr Jim Edwards of SCAS for the voiceover for the educational video.

## Author contributions

All authors were involved in the conceptualisation, design and delivery of the project. PK performed the data analysis, and PK and CF wrote the first draft of the article. All authors revised and approved the final version. CF acts as the guarantor for this article.

## Conflict of interest

None declared.

## Ethics

Not required.

## Funding

This work was funded through a Health Foundation Q Exchange project grant, 2022 cohort. The funder had no role in the design, implementation, interpretation or reporting of the project.
